# Lymphocytic interstitial pneumonia: computed tomography findings in 36 patients

**DOI:** 10.1590/0100-3984.2019.0107

**Published:** 2020

**Authors:** Guilherme Felix Louza, Luiz Felipe Nobre, Alexandre Dias Mançano, Bruno Hochhegger, Arthur Soares Souza Jr., Gláucia Zanetti, Edson Marchiori

**Affiliations:** 1 Universidade Federal do Rio de Janeiro (UFRJ), Rio de Janeiro, RJ, Brazil.; 2 Universidade Federal de Santa Catarina (UFSC), Florianópolis, SC, Brazil.; 3 Sabin Medicina Diagnóstica, Taguatinga, DF, Brazil.; 4 Universidade Federal de Ciências da Saúde de Porto Alegre (UFCSPA), Porto Alegre, RS, Brazil.; 5 Faculdade de Medicina de São José do Rio Preto (Famerp), São José do Rio Preto, SP, Brazil.

**Keywords:** Lymphocytic interstitial pneumonia, Lung cysts, Computed tomography, Pneumonia intersticial linfocítica, Cistos pulmonares, Tomografia computadorizada

## Abstract

**Objective:**

To analyze the computed tomography (CT) findings of lymphocytic interstitial pneumonia (LIP).

**Materials and Methods:**

We retrospectively reviewed the clinical and CT findings of 36 patients with LIP, including 25 women and 11 men, with a mean age of 52.5 years (age range, 22-78 years).

**Results:**

The main associated diseases with LIP were Sjögren syndrome (42%), human immunodeficiency virus infection (17%), amyloidosis (17%), Sjögren syndrome associated with secondary amyloidosis (11%), idiopathic (8%), and systemic lupus erythematosus (5%). The predominant CT abnormalities were multiple cystic airspaces (n = 35), small nodules (n = 15), ground-glass opacities (n = 13), bronchiectasis and/or bronchiolectasis (n = 8), and thickening of the bronchovascular bundles (n = 8). Other CT findings included reticular opacities (n = 7), calcified nodules (n = 4), airspace consolidation (n = 4), emphysema (n = 3), honeycombing (n = 3), lymph node enlargement (n = 2), mosaic attenuation pattern (n = 1), and cavitated nodules (n = 1).

**Conclusion:**

The main CT findings of LIP were multiple cysts, small nodules, and ground-glass opacities.

## Abstract (pt)

**Objetivo:**

Analisar os achados da tomografia computadorizada da pneumonia intersticial linfocítica.

**Materiais e Métodos:**

Trinta e seis pacientes com diagnóstico de pneumonia intersticial linfocítica (25 mulheres, 11 homens; faixa etária de 22 a 78 anos; idade média de 52,5 anos) tiveram os achados clínicos e tomográficos revisados retrospectivamente.

**Resultados:**

As principais doenças associadas à pneumonia intersticial linfocítica foram síndrome de Sjögren (42%), infecção pelo HIV (17%), amiloidose (17%), síndrome de Sjögren associada a amiloidose secundária (11%), síndrome de Sjögren idiopática (8%) e lúpus eritematoso sistêmico (5%). As alterações predominantes na TC consistiram em múltiplos espaços aéreos císticos (n = 35), pequenos nódulos (n = 15), opacidades em vidro fosco (n = 13), bronquiectasias e bronquiolectasias (n = 8) e espessamento de feixes broncovasculares (n = 8). Outros achados da TC incluíram opacidades reticulares (n = 7), nódulos calcificados (n = 4), consolidação do espaço aéreo (n = 4), enfisema (n = 3), faveolamento (n = 3), linfonodomegalia linfática (n = 2), padrão de atenuação em mosaico (n = 1) e nódulos escavados (n = 1).

**Conclusão:**

Os principais achados tomográficos observados em pacientes com pneumonia intersticial linfocítica foram múltiplos cistos, pequenos nódulos e opacidades em vidro fosco.

## INTRODUCTION

Lymphocytic interstitial pneumonia (LIP) is a benign lymphoproliferative disorder characterized by pulmonary infiltration of lymphocytes and plasma cells. It most commonly occurs in patients with Sjögren syndrome and acquired immune deficiency syndrome but can also occur in various other diseases^(1-4)^. LIP has a progressive course in over one-third of the patients and resolves after treatment with corticosteroids in almost all patients^(1,3,4)^. The clinical features and radiologic features of LIP have been described in a few isolated cases and cross-sectional studies^(2-4)^. The purpose of this study was to identify the computed tomography (CT) findings in patients with LIP.

## MATERIALS AND METHODS

### Patient selection

The institutional review board of our institution approved the study protocol and waived the requirement for patient consent. All data used in this study were anonymized. To identify patients with LIP, a search tool was used to select reports containing the term “lymphocytic interstitial pneumonia.” A total of 52 patients were identified, 21 of whom had undergone CT examination at our hospital and were therefore enrolled in the study. We also enrolled other 15 patients from eight different hospital and medical institutions in Brazil who had also undergone CT examination. Thus, we retrospectively identified 36 patients with LIP who had undergone radiologic examination between January 2008 and December 2018. The study population included 25 women and 11 men, with a mean age of 52.5 years (age range, 22-78 years). Experienced specialized pulmonologists, rheumatologists, pathologists, and radiologists had diagnosed 23 patients with LIP based on clinical, laboratory, and radiologic findings in accordance with the accepted criteria^(5-7)^. The remaining 13 patients had been diagnosed with LIP based on open lung biopsy findings.

### Image acquisition

Chest CT examinations were performed with various helical scanners, as different hospitals were involved in this study. CT acquisition parameters were 0.625-2.5-mm section thickness, 0.9-1.75 pitch, 120 kV, 80-350 mA/s or automatic tube current adjustment, and 0.6-0.8 s per gantry rotation. Image reconstruction included contiguous 1.25- or 2-mm-thick sections with high-resolution and standard algorithms for evaluating the lung parenchyma and mediastinum. Patients were examined using the single breath-hold technique.

### Image analysis

Two board-certified radiologists, both with more than 15 years of experience in chest imaging, analyzed all CT images and reached the final assessment by consensus. They were blinded to patient demographics, clinical data, and final diagnoses. All chest CT images were unenhanced. They were initially analyzed using parenchymal window settings (1200-1600 HU width; 500-700 HU level) and subsequently reviewed using mediastinal window settings (350-450 HU width; 20-50 HU level).

The readers were asked to assess the presence, extension, and distribution of cysts, small nodules, reticulation, ground-glass opacities, bronchiectasis and/or bronchiolectasis, calcified nodules, cavitated nodules, thickening of the bronchovascular bundles, airspace consolidation, emphysema, honeycombing, lymph node enlargement, among others. The criteria adopted to define these patterns were defined by the Fleischner Society and Brazilian Society of Pulmonology and Tisiology^(8,9)^.

The anatomical distribution was peripheral (subpleural) when abnormalities were predominant in the outer one-third of the lung periphery in contact with the pleural surface, and it was central when abnormalities were predominant in the inner two-thirds of the lungs in the transverse plane. In the craniocaudal direction, the lung zones were divided into the upper (abnormalities above the level of the aortic arch), middle (between the aortic arch and the carina), and lower (below the level of the carina).

LIP was diagnosed in 13 cases based on biopsy findings of peribronchiolar infiltration with lymphocyte and plasma cells and in the other 23 cases based on clinical, laboratory, and radiological findings after excluding other diseases that present with multiple pulmonary cysts, according to the accepted criteria^(5-7)^.

## RESULTS

The study population comprised 36 patients diagnosed with LIP. The causes of LIP were Sjögren syndrome, human immunodeficiency virus (HIV) infection, amyloidosis, Sjögren syndrome associated with secondary amyloidosis, idiopathic, and systemic lupus erythematosus in 15 (42%), 6 (17%), 6 (17%), 4 (11%), 3 (8%), and 2 (5%) patients, respectively.

**Figure 1:**
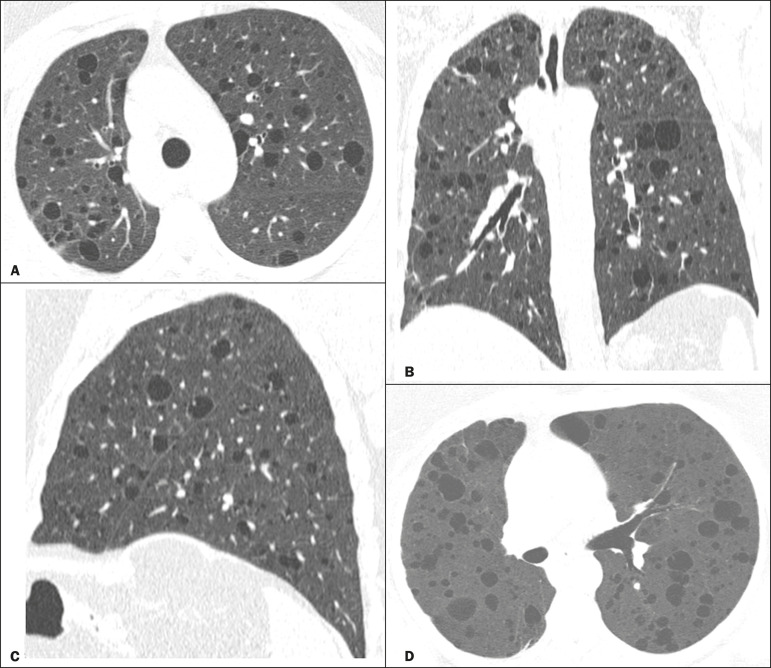
A 54-year-old woman with LIP related to Sjögren syndrome. Axial (**A**), coronal (**B**), sagittal (**C**), and minimal-intensity projection (**D**) chest CT scans show multiple bilateral pulmonary cysts with a random distribution.

**Figure 2:**
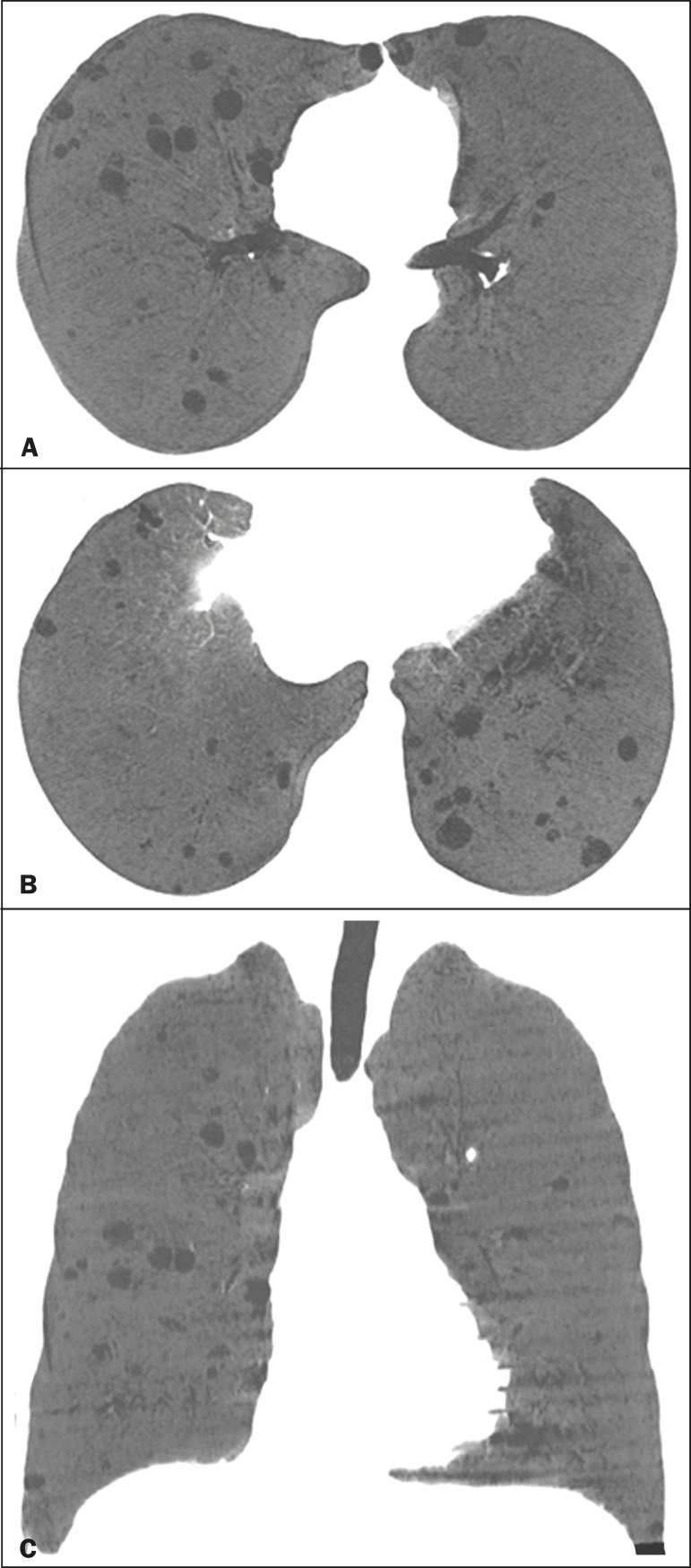
A 64-year-old woman with LIP related to Sjögren syndrome. Chest CT scans obtained with minimal-intensity projection with axial (**A,B**) and coronal (**C**) reconstructions show multiple bilateral pulmonary cysts with a random distribution.

**Figure 3:**
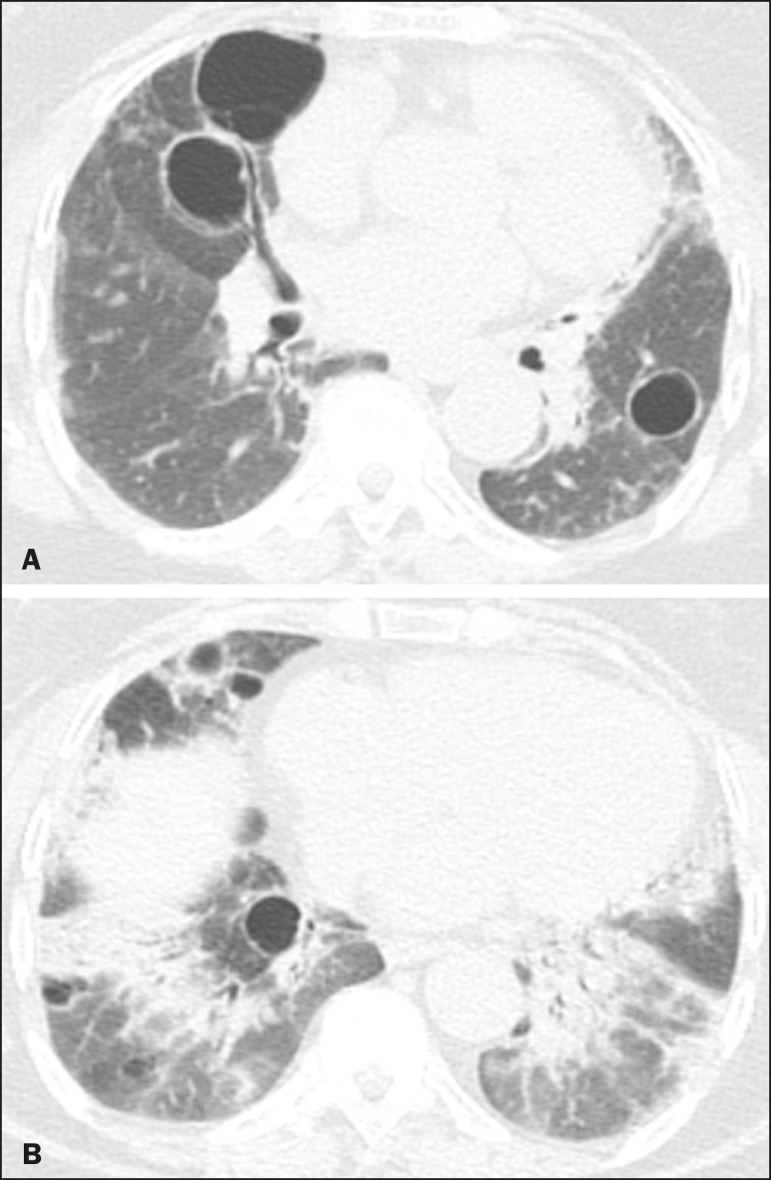
A 64-year-old man with idiopathic LIP. Chest CT scans show multiple cysts with a random and bilateral distribution associated with ill-defined ground-glass opacities and consolidations.

**Figure 4:**
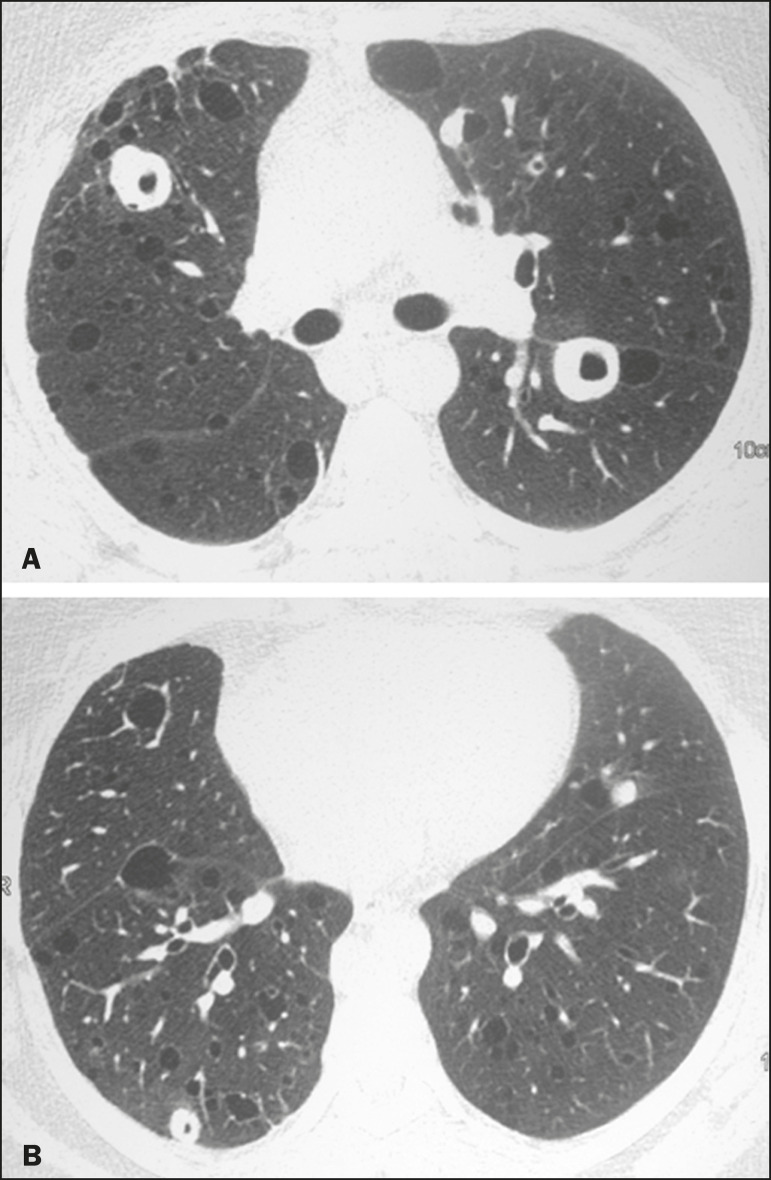
A 50-year-old woman with LIP related to amyloidosis. Chest CT scans show multiple cavitated nodules associated with pulmonary cysts.

**Figure 5:**
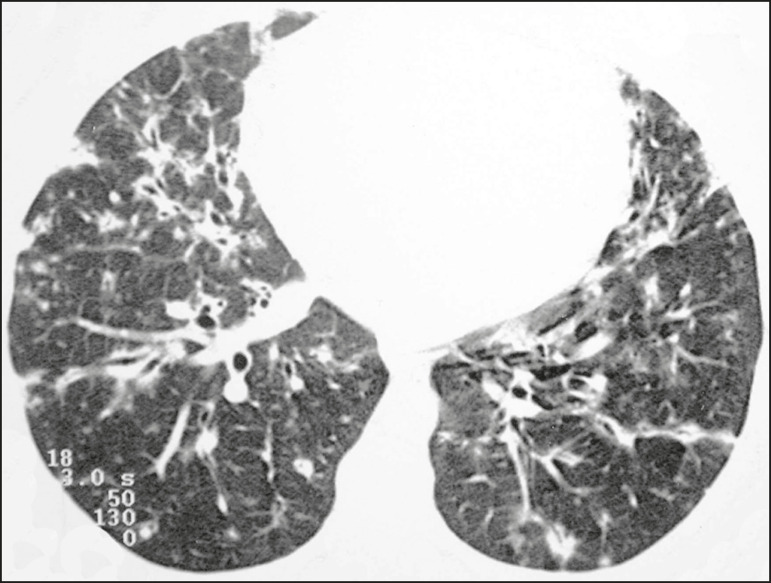
A 41-year-old woman with LIP related to Sjögren syndrome. Chest CT scan shows peribronchovascular thickening associated with multiple small nodules.

Multiple pulmonary cysts with round margins and thin walls (< 2 mm) (Figures 1-3) were present in almost all patients (n = 35; 97.2%). The number of cysts varied between 7 to 246 per examination, with a median number of 47.7 cysts per patient. The most common (49.7%) size of the cyst was 5-10 mm, followed by < 5 mm (29.2%), 11-15 mm (13.9%), 16-20 mm (4.9%), and > 20 mm (2.1%). The cystic airspaces were bilateral in 34 (94.4%) patients and unilateral in 1 (2.8%) patient. The cysts showed a random distribution in 34 (94.4%) patients and predominance in the central zones in 2 patients. Cystic airspaces were in the upper zones in 2 (5.6%) patients, in the inferior zones in 11 (30.5%) patients, and diffuse in 23 (63.8%) patients. Small nodules were present in 15 (41.7%) patients, with a bilateral distribution in 13 (86.7%) patients and unilateral distribution in 2 (13.4%) patients. The nodules were diffuse in 11 (73.3%) patients and had a patchy random distribution in 4 (26.7%) patients. The nodules were calcified in 4 patients and showed cavitation in 1 patient (Figure 4). Areas of ground-glass attenuation were present in 13 patients (36.1%), with a bilateral distribution in 12 (92.3%) patients and unilateral distribution in 1 (7.7%) patient. They had a diffuse distribution in 8 (61.5%) patients, patchy random distribution in 4 (30.7%) patients, and peripheral distribution in 1 (7.8%) patient. Bronchiectasis and/or bronchiolectasis was present in 8 (22.2%) patients, with a bilateral and patchy random distribution in all of them. One (2.8%) patient with biopsy-proven LIP presented with peribronchovascular thickening associated with multiple small nodules (Figure 5). Table 1 presents the main CT findings of LIP in this study.

**Table 1:** CT findings in patients with LIP (n = 36).

CT findings	Number of patients (%)
Pulmonary cysts	35 (97.2%)
Small nodules	15 (41.6%)
Ground-glass attenuation	13 (36.1%)
Bronchiectasis and/or bronchiolectasis	8 (22.2%)
Septal thickening	8 (22.2%)
Reticular opacities	7 (19.4%)
Calcified nodules	4 (11.1%)
Consolidation	4 (11.1%)
Emphysema	3 (8.3%)
Honeycombing	3 (8.3%)
Lymph node enlargement	2 (5.5%)
"Crazy-paving" pattern	1 (2.7%)
Cavitary nodules	1 (2.7%)

*Note:* Frequencies do not add up to 100% because some patients had more than one feature.

## DISCUSSION

LIP is an uncommon lung disease belonging to the spectrum of benign pulmonary lymphoproliferative disorders. It is a distinct clinicopathologic condition that involves inflammatory pulmonary reaction of the bronchus-associated lymphoid tissue, which culminates in cellular expansion and infiltration of the interstitium by reactive T and B lymphocytes, plasma cells, and histiocytes^(1-4,10-14)^. The main pathologic feature of LIP is dense interstitial lymphocytic infiltrates, which expand and widen the interlobular and alveolar septa. These infiltrates are typically polymorphous and composed of a combination of small lymphocytes and a variable number of plasma cells, macrophages, immunoblasts, and occasional histiocytes^(1,15,16)^. Although may be diffuse, infiltrates are predisposed to be most severe in the perilymphatic interstitium along the bronchovascular bundles, interlobular septa, and pleura^(1,3,17,18)^.

LIP affects women more frequently than men (male: female ratio, 1.00:2.75). Men are more likely to develop idiopathic LIP while women are more likely to develop LIP related to an autoimmune disorder^(1,19,20)^. In our study, a female predilection (69%) was seen both overall and in all related etiologies when cases were stratified by etiology.

LIP is most commonly described in association with Sjögren syndrome, followed by infectious causes, such as HIV and Epstein-Barr virus^(1,19,20)^. Other described causes include allogenic bone marrow transplantation^(21)^, Castleman disease^(22)^, Hashimoto disease^(23)^, myasthenia gravis^(24)^, and rheumatoid arthritis^(25)^. Associations with numerous autoimmune reactions suggest that LIP has an autoimmune pathogenic basis. Approximately 25% of the LIP cases are associated with Sjögren syndrome, and some studies estimated that 1% of the patients with Sjögren syndrome develop LIP during the course of the disease^(1,5,20)^. In our study, the prevalence of LIP related to rheumatologic diseases was also very high, reinforcing this association. The principal etiologies associated with LIP in our study were, in order of frequency, Sjögren syndrome (42%), HIV infection (17%), amyloidosis (17%), Sjögren syndrome associated with secondary amyloidosis (11%), idiopathic (8%), and systemic lupus erythematosus (5%). The relatively high prevalence of LIP related to amyloidosis in our casuistry is worth noting, as it has not been described in large cross-sectional studies^(2,3)^.

LIP generally has an insidious onset and symptom duration usually ranging from 2 months to 12 years before medical evaluation. When symptomatic, the most frequent complaints are dyspnea on exertion and nonproductive cough. Other less common symptoms include pleuritic chest pain and systemic symptoms, such as fever, weight loss, fatigue, and night sweats^(1,17-20)^.

Chest CT has been the subject of many articles recently published in the Brazilian radiological literature^(26-34)^. There have been a few large cross-sectional studies on chest CT findings in patients with LIP. In the largest one, Johkoh et al.^(3)^ described CT findings in 22 patients with biopsy-proven LIP. The main findings in that study were ground glass attenuation, poorly defined centrilobular nodules, thickening of the bronchovascular bundles, interlobular septal thickening, cystic airspaces, and lymph node enlargement. The less common findings included large nodules, emphysema, airspace consolidation, bronchiectasis, architectural distortion, honeycombing, and pleural thickening.

Our findings are in part concordant with those of Johkoh et al. study^(3)^. The main finding in our casuistry was pulmonary cysts in 97.2% of the cases. Cysts were typically bilateral, had a random and diffuse distribution in the lung parenchyma, measured 5-10 mm, and had round margins with thin walls. Johkoh et al.^(3)^ described cystic airspaces in 68% of the patients, with an average size of 6.4 ± 2.9 mm. Further, in their study, cysts had a bilateral and predominantly random distribution.

The differential diagnosis of pulmonary cystic diseases is broad^(35-38)^. The literature describes thin-walled cysts on CT as a characteristic feature of LIP, occurring in as many as 80% of the patients. Cysts have variable size and shape and are few to numerous. They usually have a random distribution, involve less than 10% of the lung, and are superimposed on ground-glass opacities. It is hypothesized that cystic airspaces may result from ischemia caused by vascular obstruction, post-obstructive bronchiolar ectasia, or bronchiolar compression as a result of peribronchiolar lymphocytic infiltration and subsegmental overinflation caused by a check-valve mechanism^(1-4)^.

Ground-glass opacities were present in all patients in the study by Johkoh et al.^(3)^ and in only 36% of the patients in our study. They were typically bilateral and diffuse in both studies. Centrilobular nodules were present in all 22 patients in the study by Johkoh et al.^(3)^ and in 41.7% of the patients in our study. The distribution of nodular opacities in our casuistic was concordant with that in Johkoh et al. study^(3)^, with the majority being bilateral and diffuse. Four patients presented with calcified nodules, all of whom had amyloidosis. In another patient with amyloidosis, nodules with cavitation were observed, probably related to amyloidosis.

The prognosis of LIP is highly variable. Some patients may have an indolent or even an asymptomatic course while others may have complications, such as associated infections, pulmonary fibrosis, and transformation into lymphoma. Spontaneous remission has been reported^(1,7,13,39)^. The management of LIP should be focused on controlling the underlying disease process. LIP is a steroid-responsive disease, with symptomatic and/or radiographic stabilization in 50-60% of the patients^(1,5)^.

Our study had some limitations. First, the study was retrospective, and analysis was cross-sectional, without the evaluation of evolutive data. The lack of clinical data limited the clinicoradiological correlation, and techniques of high-resolution CT varied with the protocol at each institution included in this study. Finally, no histopathological correlation was obtained in some patients since LIP is a well-known disease with a self-limiting course and good prognosis, and histopathological assessment in these cases lacked a reasonable risk-benefit relationship. Even in a prospective study, it would be ethically controversial to obtain histopathological diagnoses for parenchymal lesions. However, such factors did not hinder the analysis of imaging features, which was the aim of the present study. Despite these limitations, this is the largest reported series of high-resolution CT findings in patients with LIP.

In conclusion, the most frequent CT findings observed in patients with LIP were multiple cysts, small nodules, and ground-glass opacities.
